# Nitrogen Fertilization Effects on Biomass Production and Yield Components of *Miscanthus* ×*giganteus*

**DOI:** 10.3389/fpls.2017.00544

**Published:** 2017-04-18

**Authors:** Moon-Sub Lee, Andrew Wycislo, Jia Guo, D. K. Lee, Thomas Voigt

**Affiliations:** ^1^Department of Crop Sciences, University of Illinois Urbana-Champaign, UrbanaIL, USA; ^2^Energy Biosciences Institute, University of Illinois Urbana-Champaign, UrbanaIL, USA

**Keywords:** *Miscanthus* ×*giganteus*, bioenergy, biomass productivity, nitrogen fertilization, yield components

## Abstract

Grasses such as *Miscanthus* ×*giganteus* and *Panicum virgatum* (switchgrass) can potentially be used to produce bioenergy on a large scale in the Midwestern USA. The biomass productivity of these warm-season perennial grasses, particularly *M.* × *giganteus*, can be substantial, even when grown with limited inputs. The literature, however, varies regarding the nitrogen requirements for *M.* ×*giganteus* biomass production. In addition, there is a lack of information that identifies the yield-component(s) (including total tiller number, tiller weight, total tiller diameter, total tiller height, phytomer number, reproductive tiller number, vegetative tiller number, reproductive tiller height, vegetative tiller height, reproductive tiller diameter, vegetative tiller diameter, and reproductive tiller phytomer number) that contributes to *M.* ×*giganteus* biomass yields. Thus, the objective of this study was to examine the effects of fertilization on biomass yield and individual *M.* × *giganteus* plant-yield components. Plots of *M.* ×*giganteus* were planted in 2008 in Urbana, IL, USA, and received annual applications of 0, 60, or 120 kg N ha^-1^. *M.* ×*giganteus* productivity increased when nitrogen was applied; between 2011 and 2014, nitrogen applications of 60 or 120 kg N ha^-1^ produced average annual yields of 22.0 dry Mg ha^-1^ compared to 11.8 dry Mg ha^-1^ for unfertilized *M.* ×*giganteus*. Both the total number of tillers per m^2^ and the tiller weight also increased as N-application rates increased. Our results indicate that increased reproductive tiller density and tiller weight with increased N fertilization increased *M.* ×*giganteus* biomass yield.

## Introduction

When growing crops for cellulosic bioenergy, efficient production of high-yielding biomass feedstocks is a primary goal. In the U.S. Midwest, *Miscanthus* ×*giganteus* Greef et Deu ex. Hodkinson et Renvoize (hereafter *M.* ×*giganteus*), a sterile, warm-season, perennial grass, shows potential as a bioenergy crop due to its great biomass production (Heaton et al., 2008). *M.* ×*giganteus* is a rhizomatous grass native to East Asia that was first cultivated as an energy crop in Europe in the early 1980s (Lewandowski et al., 2000). It is believed to be a cross between the fertile species *M. sinensis* and *M. sacchariflorus* (Hodkinson et al., 2002). As it is sterile, *M.* ×*giganteus* must be propagated vegetatively using rhizome cuttings, rhizome-derived plugs, or *in vitro* micro propagation (Lewandowski, 1998; Anderson et al., 2011). Rhizome propagation has produced more robust plants than *in vitro* propagation (Lewandowski, 1998).

*M.* ×*giganteus* has high yield potential. In Europe, *M.* ×*giganteus* has produced 25 to 30 Mg ha^-1^ (Lewandowski et al., 2000). In the U.S., *M.* ×*giganteus* biomass productivity from University of Illinois bioenergy studies has ranged between 15 and 30 Mg ha^-1^ in several Illinois field studies (Heaton et al., 2004, 2008; Maughan et al., 2012).

Nitrogen applications to *M.* ×*giganteus* have had variable productivity results. Two long-termed *M.* ×*giganteus* fertility studies in Europe found no productivity response to N fertilization over many years (Himken et al., 1997; Christian et al., 2008), while a third study reported a N response of biomass as the plot aged beyond 10 years (Clifton-Brown et al., 2007). The Illinois *M.* ×*giganteus* studies were initially designed to compare *M.* ×*giganteus* yields with those of switchgrass (*Panicum virgatum* L.) with no added fertility (Heaton et al., 2008). As the stands aged, *M.* ×*giganteus* yields declined (Arundale et al., 2014a). However, when nitrogen was applied to the aged plots, previously unfertilized, *M.* ×*giganteus* productivity increased as the N rates increased (Arundale et al., 2014b).

Grass phenotypic traits such as tiller density, tiller length, the number of phytomers per tiller [phytomers are vegetative units of grass shoots that include an internode, leaf, a portion of the node at the upper end, and a vegetative bud and portion of the node at the lower end (Beard and Beard, 2005)], the reproductive-to-vegetative tiller ratio, and tiller weight all play a role in determining productivity in herbaceous bioenergy crops. To date, these yield components have been evaluated and correlated with biomass productivity in switchgrass and prairie cordgrass (*Spartina pectinata* Link).

A study of three switchgrass cultivars showed strong correlation with increasing yield and both tiller density and phytomer mass, and weak correlation with the number of phytomers per tiller (Boe and Beck, 2008). Similar studies also found that the number of reproductive tillers per m^2^ and the number of phytomers per tiller were good selection criteria for increased biomass production of switchgrass (Boe, 2007). In addition, Boe (2007) also reported that switchgrass plants with greater numbers of large, reproductive stems tended toward higher yields (Boe, 2007). Das et al. (2004) reported a positive correlation between yield and tiller density. Much of the overall variation in switchgrass yield, therefore, results from genetic variability among cultivars (Boe and Beck, 2008). In prairie cordgrass, another warm-season rhizomatous perennial grass, Guo et al. (2015) found that tiller mass, tiller density, heading date, plant height, and phytomer number were all positively correlated with yield in some manner, but also found that much of the phenotypic variation was from the genetic diversity of the germplasm. With respect to the yield effect of nitrogen, Muir et al. (2001) reported that switchgrass tiller mass and tiller density responded positively to increased N fertilization and that tiller mass was more important than tiller density for biomass production. Similarly, Sanderson and Reed (2000) described that high N input increased individual switchgrass tiller weight, which increased biomass production.

There are conflicting results regarding *M.* ×*giganteus* yield response to nitrogen fertilization, and the yield components that contribute to *M.* × *giganteus* biomass productivity are not well understood. Moreover, there are no reports of *M.* ×*giganteus* yield components, N-fertilizer effects on yield components, and the yield component and N fertility roles on biomass productivity. Our central hypothesis was that N fertilization will increase one or more yield components and those components will contribute to *M.* ×*giganteus* biomass. Therefore, the objective of this study was to examine the effects of fertilization on biomass yield and individual yield components in *M.* × *giganteus*.

## Materials and Methods

The study site was located near Urbana, IL, USA, at the University of Illinois Energy Farm (40.0624 N, −88.1915 W) in Dana silt loam soil (fine-silty, mixed, superactive, mesic Oxyaquic Argiudolls). Before field planting in this study, *M.* × *giganteus* rhizomes (approximately 25 g) were collected from a field nursery at the University of Illinois Landscape Horticulture Research Center (Urbana, IL, USA) in 2007, and planted into pots (9 cm × 9 cm × 12 cm) using Sunshine Metro-Mix950^®^(Sun Gro Horticulture Distribution Inc., Hadley, MA, USA) as the growing medium. The potted *M.* ×*giganteus* plants were grown in the University of Illinois greenhouse (Urbana, IL, USA) maintained at 27°C/16°C day/night temperature with 14 h photoperiod providing 400 μmol m^-2^s^-1^ photon flux at plant canopy level. In July 2008, potted *M.* ×*giganteus* were planted by hand on one-meter spacing in twelve, 10 m × 10 m plots (100 plants per plot) with three nitrogen fertility treatments applied annually in early spring at or near the time of emergence at 0, 60, and 120 kg N ha^-1^ using urea as the N source (Maughan et al., 2012). Due to winterkill during the 2008–2009 winter the site was partially replanted in spring 2009 to fill plots to 100 plants each. The study was planted using a randomized complete block design with four replications, each comprised of the three N-application levels (Maughan et al., 2012).

This study reports on 2011–2014 growing-season findings. Biomass yields in 2010 were minimal (<3 Mg ha^-1^) and data were not included in this study. From 2011 to 2014, the study was harvested post-senescence after each growing season, between mid-December and March, which is the agronomic harvest timing for *M.* ×*giganteus* grown as a bioenergy grass in Central Illinois. Biomass was cut by hand in 1-m^2^ quadrats with five replications per plot in senesced biomass harvests. Quadrats were selected throughout the plots in an attempt to produce samples representative of the plot as a whole and were not selected from border rows. Stems were cut at 10 cm and each quadrat was bundled individually. The biomass from each quadrat was measured for total plant fresh weight, subsample wet and oven-dry weights, vegetative tiller number (tiller m^-2^) and reproductive tiller number (tiller m^-2^). Five vegetative and five reproductive tillers were randomly selected from each of five replications per plot for yield components including tiller weight (g tiller^-1^), reproductive and vegetative tiller diameter (mm), reproductive and vegetative tiller height (cm), and reproductive and vegetative tiller phytomer number. Tiller diameter was measured at the midpoint of the lowest complete phytomer. Tiller height was measured to the top node of vegetative stems and to the base of the flower in reproductive stems. Dry biomass weight (PB, Mg ha^-1^) was determined by drying a 1.0 kg of subsample to 60°C for up to 72 h until dry weight was constant. Finally, we calculated nitrogen use efficiency (NUE) according to Delogu et al. (1998) and Lewandowski and Schmidt (2006), where NUE is the ratio of yield (yield at *N*_x_-yield at *N*_0_) to N supply.

Weather data including precipitation and temperature was obtained from the Illinois State Climatologist and the Illinois state water survey 2015 (Illinois State Water Survey^1^). Precipitation and temperature records are shown for the location for the duration of the study (**Table 1**).

**Table 1:** Weather conditions during 2011–2015 with 30-year average (1981–2010) for Urbana, IL, USA.

(A) Precipitation (unit: mm).
**Month**	**2011**		**2012**		**2013**		**2014**		**30-year average**	

January	17		81		65		41		48	
February	96		29		82		77		51	
March	35		41		34		35		82	
April	188		59		179		100		93	
May	125		79		95		111		122	
June	106		58		159		209		107	
July	40		15		90		221		119	
August	45		141		9		39		111	
September	69		145		17		87		82	
October	62		139		91		126		71	
November	120		27		39		61		88	
December	70		53		34		46		70	
Annual total precipitation	973		867		894		1153		1044	

**(B) Temperature (unit: °C).**

**Month**	**2011**	**2012**	**2013**	**2014**	**30-year average**	**2011**	**2012**	**2013**	**2014**	**30-year average**

January	-3.7	3.3	1.3	-3.6	-1.2	-11.7	-7.2	-8.8	-14.2	-10.2
February	0.8	4.6	1.3	-4.2	1.5	-7.3	-5.1	-7.3	-13.8	-8.2
March	9.0	17.8	3.8	5.8	8.3	-1.7	4.2	-3.0	-6.2	-2.8
April	16.2	17.2	14.3	16.1	15.4	4.3	4.1	2.6	3.6	3.4
May	20.6	25.4	22.3	22.2	21.3	9.3	11.7	10.2	9.9	9.2
June	26.8	27.8	26.0	26.4	26.4	15.5	13.6	14.3	15.8	14.9
July	31.4	33.5	26.1	24.8	27.8	19.4	18.9	15.6	13.8	16.6
August	29.4	29.0	27.3	26.4	27.1	15.9	14.4	15.0	16.3	15.6
September	22.2	22.7	26.4	22.7	24.0	10.2	10.4	12.1	10.1	10.7
October	18.4	14.6	17.2	15.7	16.8	4.2	3.6	4.7	5.0	4.2
November	10.8	8.9	7.2	4.9	8.7	0.8	-2.3	-3.6	-5.6	-1.7
December	4.4	4.9	-0.1	1.8	0.9	-3.8	-3.2	-9.4	-4.4	-7.7

*mm, millimeter; °C, degrees celsius.*

Data analysis including ANOVA, mean separation, and normality of the residuals and homogeneity of variances were performed in SAS software (SAS Institute, Cary, NC, USA). Biomass and yield components data were analyzed using Proc Mixed in SAS with N-rate (N), year (Y), and the interaction of N-rate and year (YN) were considered fixed effects and block as random. Tukey’s studentized range test was used to compare biomass yield and phenotypic traits at α = 0.05.

## Results

Monthly precipitation and temperature data for 2011–2014 are presented in **Tables 1**, **2**, respectively. June 2012 precipitation was 45% below the 30-year average at 58 mm and July 2012 precipitation was 87% below the 30-year average at 15 mm, whereas August and September 2013 were 9.1 and 9.7 mm, which are 90% less than 30-year average (**Table 1**).

**Table 2:** Probability values from analysis of variance for biomass yield and yield components^†^ of *Miscanthus* ×*giganteus* affected by N rate during 2011–2014 at Urbana, IL, USA.

	PB¶	T-TN^†^	VTN^†^	RTN^†^	TW^†^	RTD^†^	RTHT^†^	RTPN^†^	VTD^†^	VTHT^†^	VTPN^†^
N rate	0.0004	0.0015	0.1074	0.0003	<0.0001	0.0002	<0.0001	<0.0001	0.0002	<0.0001	<0.0001
Year	0.0016	<0.0001	0.0163	0.0006	<0.0001	<0.0001	0.0074	<0.0001	<0.0001	<0.0001	0.2148
N × Y	0.0204	0.0360	0.6493	0.0539	0.0003	0.0626	<0.0001	0.0133	0.6770	<0.0001	0.1830

*¶PB, plant biomass (Mg ha^-1^). ^†^T-TN, total tiller number (tiller m^-2^); VTN, vegetative tiller number (tiller m^-2^); RTN, reproductive tiller number (tiller m^-2^); TW, tiller weight (g tiller^-1^); RTTD, reproductive tiller stem diameter (mm); RTHT, reproductive tiller height (cm); RTPN, reproductive tiller phytomer number; VTD, vegetative tiller diameter (mm); VTHT, vegetative tiller height (cm); VTPN, vegetative tiller phytomer number.*

During 2011–2014, the main effects of N rate and year and their interaction effects were significant for biomass yield (**Table 2**). Biomass yield increased with increased N fertilization up to 60 kg N ha^-1^, and biomass yields between the two N fertilization rates (60 and 120 kg N ha^-1^) were not different. As interaction effects indicate, biomass yield generally appeared to decrease from 2011 to 2014 without N fertilization. However, biomass production was consistent throughout the years with N rate of 60 kg N ha^-1^ (**Table 3**). From 2011 to 2014, *M.* ×*giganteus* plots fertilized at 60 kg and 120 kg N ha^-1^ produced average annual yields of 25.5 and 24.9 Mg ha^-1^, respectively, compared to 13.0 Mg biomass ha^-1^ from the unfertilized plots (**Table 3**).

The main effects of N rate and year were significant for all biomass yield component traits except for the vegetative tiller number and vegetative tiller phytomer number, respectively, and N × year interactions were significant for total tiller number tiller weight, reproductive tiller height, reproductive tiller phytomer number, and vegetative tiller height (**Table 2**). In general, the values of all yield component traits increased with N fertilization except for the vegetative tiller number and vegetative tiller phytomer number, and differences between fertilized plots and unfertilized plots increased as the stands aged. However, no difference was observed between the two N rates (**Table 3**). In 2014, the reproductive tiller number was 24 and 42 tillers m^-2^ for 0 and 60 kg N ha^-1^, respectively, and tiller weight was 28 and 44 g tiller^-1^ for 0 and 60 kg N ha^-1^, respectively (**Table 4**). While the vegetative tiller number was not affected by N fertilization, total tiller number increased with N fertilization (**Table 3**). There was no difference in total tiller number among years, but total tiller number was lower in 2012, especially without N application (**Table 3**).

**Table 3:** *Miscanthus* × *giganteus* biomass yield¶ and yield components† as affected by N fertilization rate during 2011–2014 at Urbana, IL, USA.

**Year**	**N fertility**	**PB¶**	**T-TN^†^**	**VTN^†^**	**RTN^†^**	**TW^†^**	**RTD^†^**	**RTHT^†^**	**RTPN^†^**	**VTD^†^**	**VTHT^†^**	**VTPN^†^**
2011	0N	15.9d‡	46.5d–f	20.3	26.3	36.6cd	8.8	253.0g	13.4h	7.8	225.2c	11.3
	60N	23.3c	49.0c–f	15.2	33.8	50.0a	9.8	282.3de	14.4g	8.8	262.8ab	12.6
	120N	22.1c	53.0c–e	22.1	30.9	43.8ab	10.1	273.7ef	14.7fg	8.9	247.6b	12.3
2012	0N	11.6ef	43.7f	24.3	10.9	25.9e	8.2	237.2h	13.5h	6.6	196.6d	10.4
	60N	24.5a–c	66.9a	17.7	39.3	37.1c	9.3	304.4a–c	15.3ef	7.4	266.7a	11.8
	120N	23.7bc	68.4a	25.8	32.7	34.1cd	9.0	296.8cd	15.5de	7.2	255.6ab	11.4
2013	0N	15.3ed	48.8c–f	13.8	35.1	30.8d	8.4	258.1fg	14.3g	6.1	187.4d	10.3
	60N	28.3ab	65.3ab	13.0	52.4	43.3b	9.0	303.4a–c	16.1	7.0	266.9a	12.7
	120N	28.5a	63.9ab	15.7	48.3	44.4ab	9.3	298.8b–d	17.1b	7.1	258.3ab	12.7
2014	0N	8.46f	39.5f	14.5	25.1	21.0f	8.6	229.2h	14.6g	6.4	135.7e	9.7
	60N	25.9a–c	57.8bc	15.5	42.4	44.8ab	10.0	314.5ab	16.7bc	7.1	254.3ab	11.9
	120N	25.2a–c	54.8cd	16.0	38.8	45.6ab	10.1	315.7a	17.9a	7.1	246.7b	13.2
N rate	0N	12.8B	44.6B	18.2	24.3B	28.1B	8.5B	244.4B	13.9C	6.7B	186.2B	10.4B
Mean	60N	25.5A	59.7A	15.3	42.0A	43.6A	9.5A	301.0A	15.6B	7.6A	262.7A	12.2A
	120N	24.9A	60.0A	19.9	37.6A	43.6A	9.6A	296.2A	16.3A	7.7A	252.0A	12.4A
Year	2011	20.4b	49.5b	19.2ab	30.3bc	42.4a	9.6a	270.0b	14.1d	8.5a	245.2a	12.1
Mean	2012	19.9b	59.7a	22.6a	27.6c	32.4c	8.8b	279.4a	14.8c	7.1b	239.6a	11.2
	2013	24.0a	59.3a	15.3b	45.2a	39.5b	8.9b	287.0a	15.8b	6.7c	237.5a	11.9
	2014	19.9b	50.7b	14.1b	35.4b	37.1b	9.5a	286.3a	16.4a	6.9bc	212.2b	11.6

*¶PB, plant biomass (Mg ha^-1^). †T-TN, total tiller number (tiller m^-2^); VTN, vegetative tiller number (tiller m^-2^); RTN, reproductive tiller number (tiller m^-2^); TW, tiller weight (g tiller^-1^); RTTD, reproductive tiller stem diameter (mm); RTHT: reproductive tiller height (cm); RTPN: reproductive tiller phytomer number; VTD, vegetative tiller diameter (mm); VTHT, vegetative tiller height (cm); VTPN, vegetative tiller phytomer number. ‡Value with the same letter with in each of interaction effect of N rate and Year, main effect of N rate, and main effect of Year are not significantly different as indicated by HSD test at P = 0.05 level.*

**Table 4:** Correlation coefficients between biomass yield and yield components^†^ of *M.* × *giganteus* during 2011–2014 at Urbana, IL, USA.

Year	T-TN^†^	VTN^†^	RTN^†^	TW^†^	RTD^†^	RTHT^†^	RTPN^†^	VTD^†^	VTHT^†^	VTPN^†^
2011	0.80^∗∗^	0.30	0.56	0.77^∗∗^	0.77^∗∗^	0.41	0.33	0.80^∗∗^	0.56	0.54
2012	0.94^∗∗^	-0.44	0.95^∗∗^	0.93^∗∗^	0.86^∗∗^	0.92^∗∗^	0.86^∗∗^	0.79^∗∗^	0.90^∗∗^	0.67^∗^
2013	0.96^∗∗^	0.23	0.95^∗∗^	0.93^∗∗^	0.89^∗∗^	0.84^∗∗^	0.78^∗∗^	0.74^∗∗^	0.87^∗∗^	0.88^∗∗^
2014	0.92^∗∗^	0.15	0.97^∗∗^	0.97^∗∗^	0.63^∗∗^	0.95^∗∗^	0.90^∗∗^	0.83^∗∗^	0.96^∗∗^	0.49

*^∗^Coefficient of correlation significant at P < 0.05. ^∗∗^Coefficient of correlation significant at P < 0.01. ^†^T-TN, total tiller number (tiller m^-2^); VTN, vegetative tiller number (tiller m^-2^); RTN, reproductive tiller number (tiller m^-2^); TW, tiller weight (g tiller^-1^); RTTD, reproductive tiller stem diameter (mm); RTHT, reproductive tiller height (cm); RTPN, reproductive tiller phytomer number; VTD, vegetative tiller diameter (mm); VTHT, vegetative tiller height (cm); VTPN, vegetative tiller phytomer number.*

**FIGURE 1:**
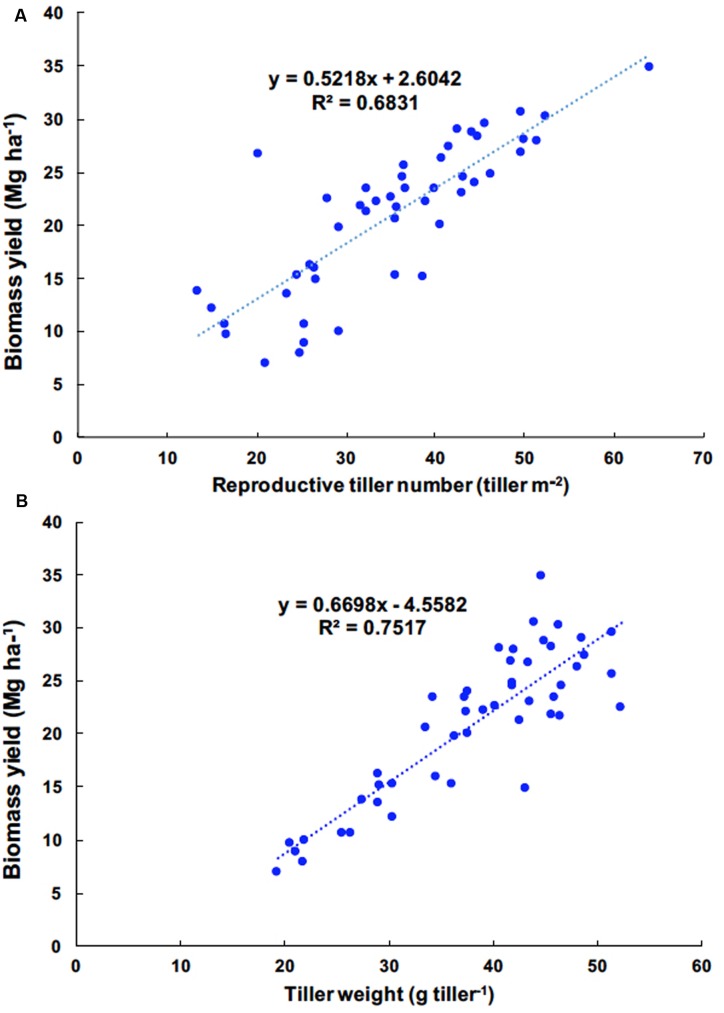
**Linear regression of reproductive tiller number and biomass yield (A)** and tiller weight and biomass yield **(B)** of *Miscanthus* ×*giganteus* fertilized by N rates, 0, 60, and 120 kg N ha^–1^ during 2011–2014 at Urbana, IL, USA.

The correlations between yield components and biomass yield in 2012, 2013, and 2014 were highly significant, exclusive of vegetative tiller number and vegetative tiller phytomer number in 2014 (**Table 4**). In 2011, there were weak, or no, observed correlations between yield components and biomass yield. Among biomass yield components, total tiller number, reproductive tiller number, and tiller weight were positively correlated with biomass yield and were the strongest indicators for biomass yield (**Table 4**). When correlation analysis between yield components and biomass yield were performed across years, the highest correlations were observed between reproductive tiller number and biomass yield (*R*^2^ = 0.6831) and tiller weight and biomass yield (*R*^2^ = 0.7517) (**Figures 1A,B**, respectively).

## Discussion

In this experiment, nitrogen fertilization increased *M.* ×*giganteus* productivity during 2011–2014, but there were no yield differences between plots fertilized with 60 and 120 kg N ha^-1^. The positive responses to N fertilization are in agreement with Arundale et al. (2014b). Furthermore, N management is particularly essential for a biomass feedstock because N is associated with productivity and the cost of production (Vogel et al., 2002). The NUE was 0.3 Mg (kg N)^-1^ for the 60 kg N ha^-1^ treatments and 0.1 Mg (kg N)^-1^ for the 120 kg N ha^-1^ treatments. Increased N application rate led to a reduction in NUE, which is similar to the finding of Lewandowski and Schmidt (2006). However, many other studies reported that N fertilization is not required to achieve high *M.* ×*giganteus* biomass yields (Himken et al., 1997; Lewandowski et al., 2000; Heaton et al., 2004; Christian et al., 2008). The different responses to N applications can be explained by the following: (1) Much of the *M.* ×*giganteus* productivity research was conducted in Europe, and despite of the spatial variations, generally atmospheric N deposition rates are higher in Western Europe than in the USA (Holland et al., 2005). The topographical difference might affect soil N, which is thus related to N fertilization biomass yield response. (2) The reported absence of N fertilization effect could also be attributed to relatively short-termed experiments or to *M.* ×*giganteus* growth during establishment years (Miguez et al., 2008). To produce 15 Mg ha^-1^ of *M.* ×*giganteus* biomass, the N, P, and K requirements would be 92 kg N ha^-1^, 13 kg P ha^-1^, and 204 kg K ha^-1^ based on yearly crop off-take (Beale and Long, 1997). In addition, in Ercoli et al. (1999), it was implied that if N fertilizer was not supplied to the cropping system, there would be a reduction of biomass yield over long-termed growth. Conversely, if *M.* ×*giganteus* is continuously harvested, there is N removal from the soil that should be compensated for by an external source of N. (3) Soil type, especially soil texture, can be an important factor for soil N availability. Even though the soil in our plots was classified as a silt loam soil by the USDA Soil Survey, based on our soil analysis (Maughan et al., 2012), this soil was a sandy loam soil with low CEC and N content. Biomass yield response to N fertilization in our study could be associated with low soil N retention as we observed no yield differences among N-fertilized plots during 2009–2010 (data not shown). Our results suggested that site-specific N management is necessary for sustainable biomass production of *M.* × *giganteus.*

Precipitation is the most important factor that directly and indirectly impacts aboveground biomass production in terrestrial ecosystems (Kardol et al., 2010), and roots are the primary connection between soil and soil water to plants (Clothier and Green, 1997; Xi et al., 2013). Plant biomass production positively responds to annual precipitation (Paruelo et al., 1999), and the seasonal precipitation pattern is a key factor in determining perennial grass establishment and biomass yield (Lee and Boe, 2005; Anderson et al., 2015). In addition, Richter et al. (2008) showed that growing season (April–September) precipitation and soil moisture capability are critical factors for perennial grass biomass production. Even though *M.* ×*giganteus* is a warm-season, C_4_ grass with high water-use efficiency, biomass productivity can be affected by precipitation during the April–September growing season (Heaton et al., 2004). Anderson et al. (2011), found that *M.* ×*giganteus* has little drought tolerance or the ability to cope with environments that receive limited precipitation. *M.* × *giganteus* roots have grown to an approximate depth of 1.8 m (Carroll and Somerville, 2009), and Neukirchen et al. (1999) reported that *M.* ×*giganteus* produced 28% of total root biomass in the top 0.30 m soil depth with nearly 50% of the total roots growing in soil layers deeper than 0.90 m. Moreover, Chimento and Amaducci (2015) reported that roots of herbaceous crops, including giant reed, switchgrass and *M.* ×*giganteus*, had more than 50% of the whole root biomass in the 30 cm of soil, and specifically, a substantial portion of *M.* ×*giganteus* roots, including fine root biomass and root length density, was distributed in the upper soils. Conversely, Monti and Zatta (2009) wrote that compared to switchgrass where 35% were found in the upper 0.35 m soil, nearly 90% of total *M.* ×*giganteus* roots were found in that soil layer.

With regard to N fertilization and water availability, Chimento and Amaducci (2015) reported that switchgrass root biomass was greater than that of giant reed, and Amaducci et al. (2017) found that switchgrass biomass production was impacted by water availability in fertilized plots, but not in unfertilized plots. Water availability affected the biomass yield of giant reed (*Arundo donax* L.) in both unfertilized and fertilized plots (Amaducci et al., 2017). Therefore, switchgrass had higher root biomass production than giant reed (Chimento and Amaducci, 2015), which resulted in less sensitivity to water availability than giant reed (Amaducci et al., 2017). On the other hand, Mann et al. (2013) wrote that switchgrass roots are likely to stretch deeply into areas of available soil moisture to overcome increasing moisture deficits that take place near the surface. In this experiment, the precipitation was variable during the 4-year time study period with much less precipitation than the 30-year average during June and July 2012 (32% of the 30-year average) and August and September 2013 (10% of 30-year average). We observed that *M.* ×*giganteus* biomass yields declined in the unfertilized plots in 2012, while there were no yield reductions in the fertilized plots. It is possible that *M.* ×*giganteus* tends to adopt a tolerance strategy (Lambers et al., 2008; Farooq et al., 2009) by relying on shallow rhizome production rather than mining deep wet soils. Limited rooting and root production in unfertilized *M.* ×*giganteus* may have limited biomass production during dry growing seasons. Even though *M.* ×*giganteus* is likely to exploit shallow rhizome production to overcome water-deficient conditions (Mann et al., 2013), applying N fertilization may help *M.* ×*giganteus* to develop root structures, which may increase potential water uptake from the subsoil, and thereby overcome periods of low water availability in topsoil (Smika et al., 1961; Viets, 1962; Neukirchen et al., 1999). In addition, it has been reported that drought tolerance in plants could be enhanced by increased N fertilization (Halvorson and Reule, 1994; Fife and Nambiar, 1997; Van Schaik et al., 1997). For instance, N fertilization may alleviate drought stress by preventing cell membrane damage and improving osmoregulation (Saneoka et al., 2004).

Nitrogen fertilization is important for tiller, tiller density, and panicle development as well as for seed production in perennial grasses (Canode and Law, 1978; Haferkamp and Copeland, 1984; Thompson and Clark, 1989, 1993). In this study, N fertilization increased the total number of tillers and the ratio of reproductive tillers and vegetative tillers which resulted in increased tiller weight and biomass yield. This finding agrees with the results reported for switchgrass by Sanderson and Reed (2000) and Muir et al. (2001). Nitrogen fertilization increased tiller survival and N deficiency during early stages of tiller development seemed particularly unfavorable to tiller survival (Power and Alessi, 1978). For example, on average, *M.* ×*giganteus* expanded vegetatively 0.15 m year^-1^ and tiller density within the center of a clone decreased as stands age, while tiller density increased toward the clone exterior (Matlaga et al., 2012). Therefore, enhanced N uptake, resulting from N-fertilization, may supply adequate amounts of various nutrients to individual tillers to ensure development and activation of the essential enzyme systems necessary for tiller survival and growth (Power and Alessi, 1978).

Pearson correlation coefficients revealed strong relationships between yield components and biomass yields (**Table 4**) and strong linear relationships occurred between biomass yields and total number of tillers, reproductive tillers, and tiller weights. Boe and Beck (2008) described that strong linear relationships have been observed between biomass yields and tiller density (tiller m^-2^) and tiller weight (mass tiller^-1^) in switchgrass. Das et al. (2004) suggested that tiller density per plant can be used as an indirect selection trait for increasing biomass yield, which can be applicable for *M.* ×*giganteus*. Moreover, no relationship was found between biomass yields and the number of vegetative tillers, while the number of reproductive tillers was highly correlated with biomass yields, implying that as reproductive tiller increased, biomass yield also increased. With regard to reproductive tillers, Boe and Casler (2005) reported that biomass produced by high-yielding switchgrass cultivars contained predominately reproductive tillers with the maximum number of phytomers tiller^-1^, and low-yielding types mostly made up of a large number of vegetative tillers having fewer phytomers and lower weight phytomer^-1^ than reproductive tillers. Boe and Casler (2005) also wrote that switchgrass biomass yields at Madison, WI, USA, were much higher than at Brookings, SD, USA, with the differences resulting from the number of reproductive tillers; the reproductive tillers were approximately three times heavier than the vegetative tillers for cultivars of switchgrass across several environments. In this experiment, the total number of tillers between fertilized and unfertilized plots was significantly different, whereas the number of vegetative tillers was not affected by N fertilization. In addition, adding N fertilization led to an increased number of reproductive tillers, and a correlation between reproductive tiller numbers and biomass yield increased over years. Wilkins (1995) reported that the application of N resulted in the portion of reproductive tiller in perennial ryegrass. This indicates that the increased total tiller number resulted from an increase in reproductive tiller number over consecutive N applications, which ultimately increased the biomass yields.

There has been substantial interest in *M.* ×*giganteus* as a bioenergy feedstock due to its high yield potential. Results from our 4-year field evaluation suggest that N fertilization might be necessary for sustainable biomass production with 60 kg N ha^-1^ being potentially adequate for maximum biomass yield. Nitrogen fertilization is necessary to maintain the tiller density and reproductive development, which are critical yield components for *M.* ×*giganteus* biomass production. These findings indicate that determining optimal agronomic management could be a useful tool for improving *M.* ×*giganteus* biomass yields.

## Author Contributions

M-SL, JG, and DL: Contributing substantial data analysis and interpretation for the work; revising the work for important intellectual content; approving final version to be published; and agreeing to be accountable. AW: Contributing substantial data acquisition, analysis, and interpretation for the work; drafting the work and revising the work critically for important intellectual content; approving final version to be published; and agreeing to be accountable. TV: Contributing substantial conception and design of the work; data analysis and interpretation for the work; revising the work for important intellectual content; approving final version to be published; and agreeing to be accountable.

## Conflict of Interest Statement

The authors declare that the research was conducted in the absence of any commercial or financial relationships that could be construed as a potential conflict of interest.

## Footnotes

1 http://www.isws.illinois.edu
